# Vascularized Nerve Flap for Spinal Cord Repair—A Preliminary Study

**Published:** 2011-03-16

**Authors:** Kazuki Kikuchi, Hidehiko Yoshimatsu, Makoto Mihara, Mitsunaga Narushima, Takuya Iida, Isao Koshima

**Affiliations:** Department of Plastic Surgery, University of Tokyo, Tokyo, Japan

## Abstract

**Object:** By making the “vascularized nerve flap for complete spinal cord gap” model, we assessed the histological effect of this method. **Method:** Eight female Sprague-Dawley rats were divided into 2 groups: group A and group B. Group A included “nonvascularized nerve flap” models, whereas group B consisted of “vascularized nerve flap” models. In each group, we transferred peripheral nerves to fill the void made by complete surgical transection of the spinal cord. Postoperatively, we administered intravenous antibiotics and performed urethral catheterization and disimpaction everyday. Tissue sampling was done on postoperative day 10. **Results:** No necrosis occurred in group B, vascularized models. In group A, nonvascularized models, necrosis of the grafted nerves was observed in 2 of the 4 rats. As for the specimens of Kluver-Barrera's method for selective myelin sheath staining, in group B, all specimens were stained with Kluver-Barrera's method for selective myelin sheath staining, whereas in group A, 2 specimens with successful engraftment were not stained. With type IV collagen immunostaining, the increase of type IV collagen around the grafted area was not so severe, compared with that of normal region in vascularized cases. In nonvascularized cases, the appearance of type IV collagen was prominent. Therefore, in the vascularized cases, it was made clear that Schwann cells survived in vascularized models and that fibrotic scar formation was inhibited. **Conclusion:** Histological comparison between vascularized and nonvascularized peripheral nerve transfer for complete spinal cord gap showed significant differences in 10 days. A longer follow-up period is necessary for observation of functional differences.

We have performed vascularized nerve transfer in many cases of peripheral nerve damage. Applying this method on spinal cord repair, we conducted a preliminary study and assessed its validity, using rats.

The total number of patients with spinal cord injuries in Japan, including congenital spina bifida or myelomeningocele and myeloschisis, is currently estimated at approximately 100000. It is estimated that approximately 5000 persons are newly affected every year. Although the regeneration of injured spinal cord and restoration of function are desires common to patients with spinal cord injuries all over the world, a fundamental treatment has not yet been established. Recently, fetal spinal cord tissue, Schwann cells (glial cells of the peripheral nervous system), olfactory ensheathing cells, and various stem cells, including neural stem cells, bone marrow stem cells, and induced pluripotent stem cells, have been transplanted into experimental animal models.^1^ A series of reports of achieving regeneration and functional restoration of the spinal cord after transplantation of a variety of these tissues and cells attracted much attention. Proper spatial arrangement of nerve cells and glial cells is required to regenerate tissues of the spinal cord; surgical techniques to allow this arrangement have not been developed yet.

In this study, we established and performed a novel transplant, vascularized peripheral nerve transfer (autologous transplantation), in rats using “super-microsurgery.” We then histologically assessed the effect of these transplants on spinal cord injuries. In peripheral nerve transfer, vascularized nerves have been known to produce an accelerated elongation of regenerated axons compared with nonvascularized nerves.^2^ Transplantation of vascularized nerve flaps using microsurgery has been found to achieve better nerve regeneration, even in cases of a longer nerve defect and with poor blood flow at the recipient site compared with free nerve transplants, since the survival rate of Schwann cells is almost 100% in these vascularized nerve flaps. In this study, we examined the application of this technology and concept to the central nervous system.

The roles of Schwann cells in peripheral nerve repair are as follows: (1) they create myelin sheath and help the axonal conduction, (2) they increase and migrate to be a scaffold for regenerating nerves, and (3) they produce neurotrophic factors. In the vascularized graft, survival rate of Schwann cells is nearly 100%, and a sufficient blood circulation can prevent the fibrotic scar formation.

Since the 1990s, a variety of inhibitory agents in central nerve regeneration were discovered. Many studies on attempts to regenerate nerves in central nervous system were done by inhibiting reactions at each stage of tissue repair cascade. Recently, fibrotic scar formation is reported to play a primary role in the inhibition of central nerve repair. Vascularized peripheral nerve transfer was suggested to be an effective method for inhibition of fibrotic scar. In 1996, much attention was paid to a report by Cheng et al^3^ published in *Science Magazine*, in which axon growth and functional restoration were observed after bridging spinal cord gaps with a fascicle of intercostal nerves in rats; this procedure is, however, complicated and further study is not yet performed. Inspired by the report, we performed peripheral nerve flap transfer for the spinal cord gap. In accordance with other studies on central nerve regeneration, tissue sampling was done on postoperative day 10. This is thought to be the shortest duration after which central nerve regeneration begins. We observed significant effects of the vascularized nerve transfer for spinal cord repair, although the study is still at a preliminary stage. A longer follow-up period is needed to observe functional differences.

In the future, we plan to pursue the possibility of the vascularized spinal cord transfer as an innovative approach to the treatment of spinal cord damage, especially ones caused by tumors (eg, myelomeningocele) and ruptured spines.

## METHODS

**Figure 1:**
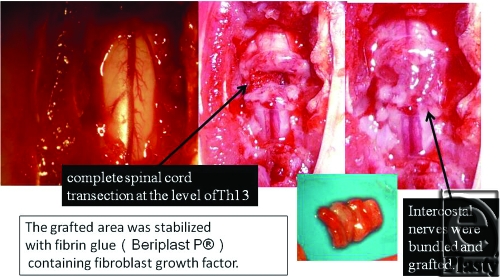
Peripheral nerve transfer for spinal cord (nonvascularized). Complete spinal cord transection was made at the level of Th 13, and then intercostal nerves were bundled and grafted. The grafted area was stabilized with fibrin glue (Beriplast P) containing fibroblast growth factor.

**Figure 2:**
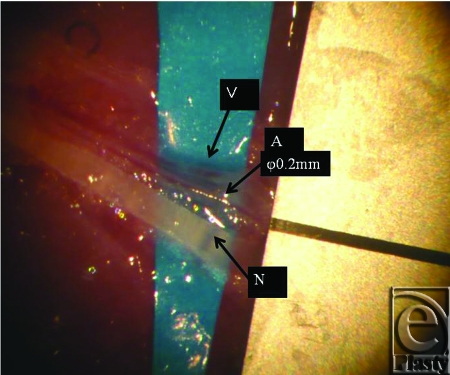
The intercostal artery (A), vein (V), and nerve (N) of rats.

**Figure 3:**
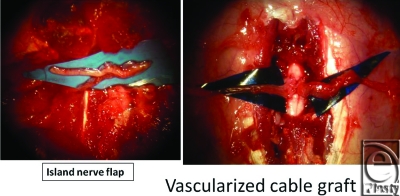
Vascularized nerve flap for complete spinal cord gaps. The 2 island flaps were folded and bridged to the spinal cord gap. The flaps were fixed with 11–0 nylon sutures.

First, we conducted a study similar to that of Cheng et al.^3^ Next, we made another group of the vascularized nerve flap transfer. The purpose of this study was to make histological comparison between vascularized and nonvascularized peripheral nerve transfers by making the “vascularized nerve flap” model. Eight female Sprague-Dawley rats were divided into 2 groups: group A and group B. Group A included “nonvascularized nerve flap” model, the same model used in the study by Cheng et al. Group B consisted of “vascularized nerve flap” model. A spinal cord segment was completely removed after laminectomy under deep anesthesia. A sharp scalpel blade was used to transect spinal cords. In group A, complete spinal cord transection was made at the level of Th 13, before intercostal nerves were bundled and grafted. The grafted area was stabilized with fibrin glue (Beriplast P) containing fibroblast growth factor (Fig 1). In group B, which comprised vascularized models, we harvested island intercostal nerve flaps bilaterally at the same level (Th 13). The nerve flaps were vascularized by the intercostal artery and vein. The diameter of the artery was approximately 0.2 mm (Fig 2). The 2 island flaps were folded and bridged to the spinal cord gap. The flaps were fixed with 11–0 nylon sutures. From its appearance, we named this flap “vascularized cable graft” (Fig 3). Postoperatively, intravenous antibiotics were administered every day, and daily urethral catheterization and disimpaction were also performed. Tissue sampling was done on postoperative day 10.

## RESULT

**Figure 4:**
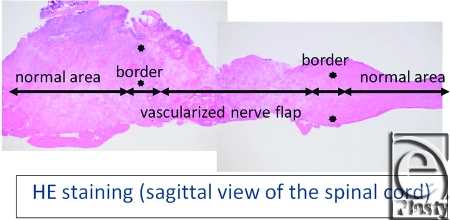
In the HE staining of group B, sagittal view of the spinal cord showed that the vascularized nerve flap was grafted successfully (original magnification; ×4).

**Figure 5:**
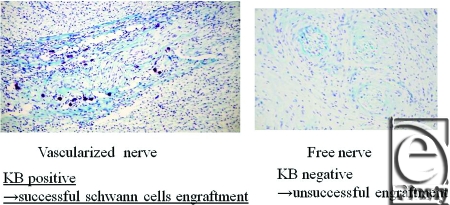
Kluver-Barrera's method for selective myelin sheath staining. In vascularized cases, all showed positively stained image, and in nonvascularized cases, specimens from the 2 successful engraftment cases showed negatively stained image. Photomicrograph of sections of 1-µm thickness that were cut from the segments and stained. Four specimens of group A were KB positive, but 2 specimens of group B were KB negative (original magnification; ×20).

**Figure 6:**
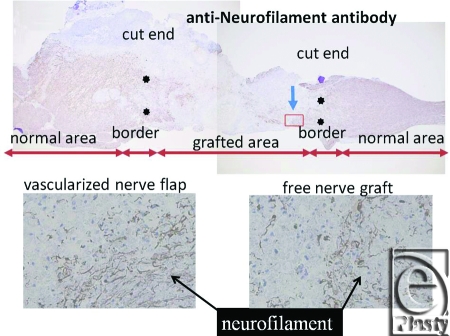
Entire areas including grafted nerves and the coaptation sites were carefully harvested and prepared for longitudinal sectioning. At the distal coaptation sites (blue arrow), neurofilament ingrowth from the cord into the graft was observed and compared between the 2 groups. This immunostaining for antineurofilament antibody showed slight axonal sprouting, but there was no significant difference between the 2 groups (original magnification; ×4).

**Figure 7:**
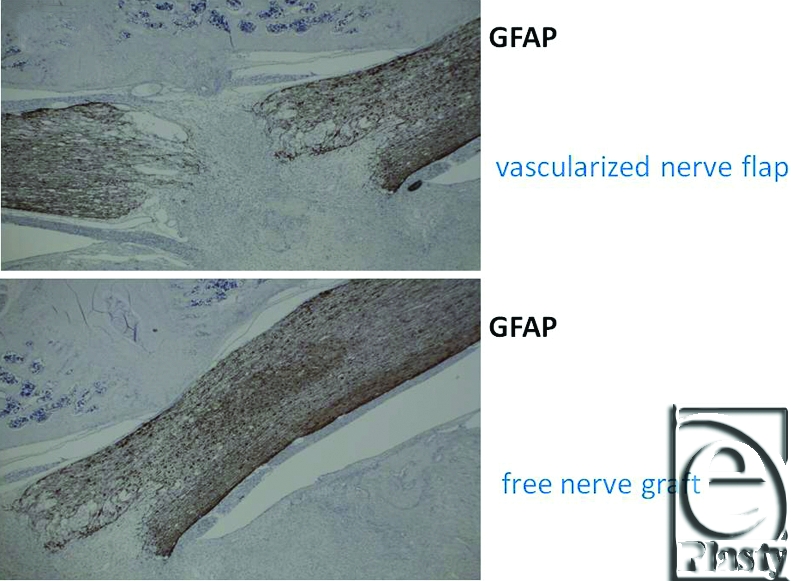
In glial fibrillary acidic protein immunostaining, glial scar formation occurred around the grafted area in both groups (original magnification; ×2).

**Figure 8:**
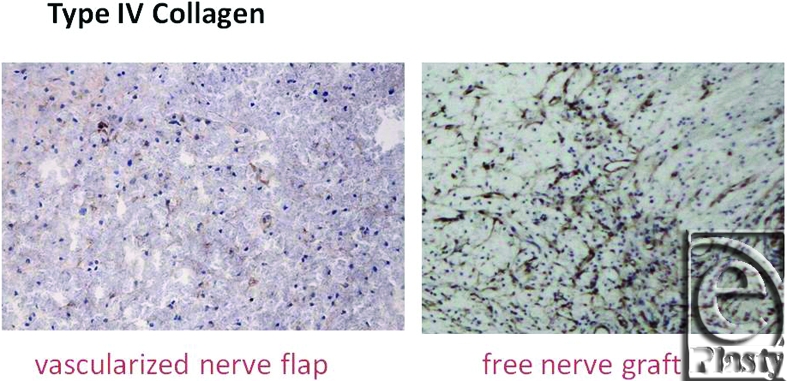
To evaluate fibrotic scar formation in the coaptation sites, nerve segments of 2 mm were harvested from the distal coaptation sites and sectioned longitudinally. In the 2 specimens of nonvascularized cases, the appearance of type IV collagen was prominent. On the contrary, the 4 specimens of the vascularized cases did not stain well by this immunostaining (original magnification; ×10).

**Figure 9:**
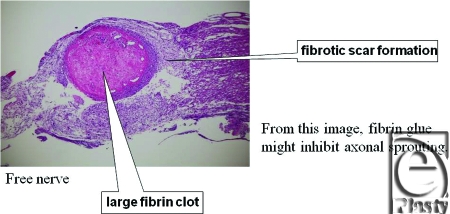
Following Cheng et al's procedure, in group A, we used fibrin glue to fix the grafts, but it produced fibrin clots and was surrounded by fibrotic scar. From this image, fibrin clots might inhibit axonal sprouting (original magnification; ×4).

**Figure 10:**
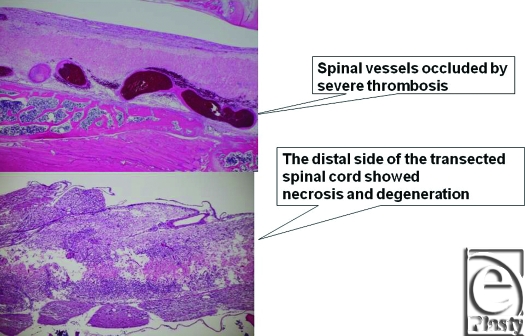
A drawback of this spinal cord injury model. Spinal vessels were occluded by severe thrombosis, and the distal side of the transected spinal cord showed necrosis and degeneration (original magnification; ×2).

Macroscopically, in vascularized cases (group B), no necrosis occurred, but in nonvascularized cases (group A), necrosis of grafted nerves was observed in 2 of the 4 rats. In group B, sagittal view of hematoxylin-eosin (HE) staining of the spinal cord showed that the vascularized nerve flap was grafted successfully (Fig 4). As for the specimens of Kluver-Barrera's method for selective myelin sheath staining (KB staining), in vascularized cases, all showed positively stained image, and in nonvascularized cases, specimens from 2 successful engraftment cases showed negatively stained image. These specimens of KB staining were made from nerve segments of grafted nerves. Specimens cut from the segments were 1 µm thick. Four specimens from group A were KB positive, but 2 specimens from group B were KB negative (Fig 5). Despite the macroscopic similarities, KB staining clarified a histological difference between group B and the 2 successful engraftment cases in group A; regarding Schwann cell engraftment, group B was successful and group A was a failure. In group B, Schwann cells survived much longer than in group A. Immunostaining of antineurofilament antibody showed slight axonal sprouting, but there was no significant difference between the 2 groups. Entire areas including grafted nerves and the coaptation sites were carefully harvested and prepared for longitudinal sectioning. At the distal coaptation sites (blue arrow) neurofilament ingrowth from the cord into the graft was observed and compared between the 2 groups (Fig 6). A longer follow-up period is considered to be necessary for the observation of any histological difference. In glial fibrillary acidic protein immunostaining, glial scar formation occurred around the grafted area in both groups (Fig 7). To evaluate fibrotic scar formation in the coaptation sites, 2 mm of nerve segments were harvested from the distal coaptation sites and sectioned longitudinally. Type IV collagen immunostaining confirmed that in vascularized cases, the increase of type IV collagen around the grafted area was not so significant compared with that in normal area. In 2 specimens of nonvascularized cases, the appearance of type IV collagen was prominent; the increase of type IV collagen was inhibited only in the vascularized cases. Four specimens of the vascularized cases were not stained sufficiently by this method (Fig 8). Therefore, in the vascularized cases, it was made clear that Schwann cells survived well in the grafted nerves and fibrotic scar formation between the spinal cord and the grafted nerves was inhibited. Following Cheng et al's procedure, in group A, we used fibrin glue to fix the grafts, but it produced fibrin clots and was surrounded by fibrotic scar. The HE staining image suggests that fibrin glue might be inhibiting axonal sprouting (Fig 9). A setback of this spinal cord injury model stems from complete segmental removal of the cord. Spinal vessels were occluded by severe thrombosis, and histologically, the distal side of the transected spinal cord showed necrosis and degeneration (Fig 10).

## DISCUSSION

For nearly 100 years, it has been considered true that central nerve does not regenerate. Recently, especially since 1990s, a variety of inhibitory agents in central nerve regeneration have been identified. And the relationship between fibrotic scar inhibition and central nerve regeneration has been focused on for many years. More than 50 years ago, it was already reported that the inhibition of glial scar by drugs makes central nerve regeneration possible.^4^ Teng et al^5^ of the Tokyo Metropolitan Institute for Neuroscience reported that infusion of dipyridyl, iron chelator, just after nerve tract transection promoted central nervous axonal sprouting beyond the lesion by inhibiting fibrotic scar formation. Kawano et al^6^ also used olfactory ensheathing cells to inhibit the fibrotic scar formation. They transected the spinal cord with a surgical knife (did not remove it but only made an incision in it) to induce spinal cord injury, immediately after which they transplanted 150000 olfactory ensheathing cells into the lesion site. Ten days later, the site was immunostained and examined. They reported that fibrotic scar formation was prevented, and transected dopaminergic axons were regenerated beyond the site of the lesion.^4^ The inhibition of fibrotic scar might be a breakthrough in central nerve regeneration.

Finally, as future prospects, we plan to design a vascularized spinal cord flap.

**Figure 11:**
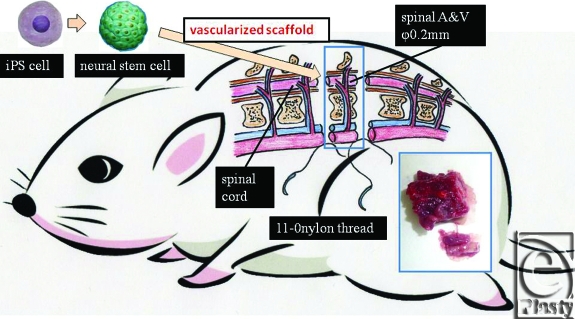
Future prospects; vascularized spinal cord transfer. A variety of cell transplantations were reported, but the proper spatial arrangement of nerve cells and glial cells is required to regenerate tissues of the spinal cord, and there is an issue that the technologies for achieving this have not been developed. This vascularized spinal cord flap can provide solutions to issues surrounding the regenerative medicine in terms of the vascularized scaffold.

**Figure 12:**
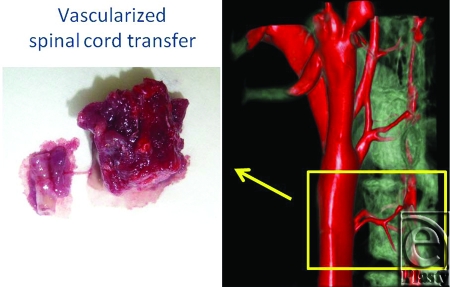
The vascularity of rats' spinal cords examined by micro computed tomographic system.

**Figure 13:**
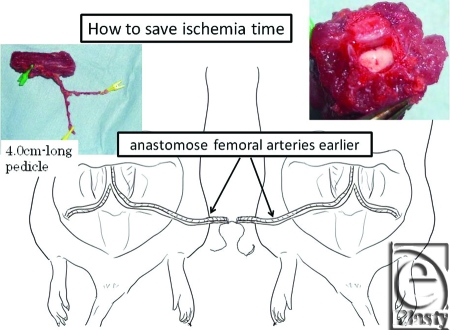
Our ingenuity was employed to eliminate ischemia. Anastomosis between the donor's femoral artery and recipient's femoral artery allowed the establishment of retrograde blood flow in the tissue transplant, preserving the blood flow in the transplanted tissues even when the aorta was clamped. Therefore, anastomosis of the aorta and vena cava could be performed separately while confirming that no ischemia was present.

A variety of cell transplantations were reported, but the proper spatial arrangement of nerve cells and glial cells is required to regenerate tissues of the spinal cord, and there is an issue that the technologies for achieving this have not been developed. The vascularized spinal cord flap can provide solutions to issues surrounding the regenerative medicine in terms of the vascularized scaffold (Fig 11). We examined the vascularity of rats' spinal cords with micro computed tomographic system (Fig 12). In the near future, a hybrid method in which vascularized spinal cord flap transfer is performed with a variety of cell transplantations is predicted to come into practice. This procedure cannot be done successfully with ischemic period. We have already developed a spinal cord transplant model without ischemic period. Anastomosis between the donor's femoral artery and recipient's femoral artery allowed the establishment of retrograde blood flow in the tissue transplant, preserving the blood flow in the transplanted tissues even when the aorta was clamped. Therefore, anastomosis of the aorta and vena cava could be performed separately while confirming that no ischemia was present (Fig 13).

In this study, we assessed the effect of the vascularized peripheral nerve transfer for complete spinal cord gap. Histological comparison between vascularized and nonvascularized peripheral nerve transfers for complete spinal cord gap revealed significant differences in 10 days. A longer follow-up period is necessary for observation of functional differences. Finally, by modifying this experimental protocol, we will explore the further possibility of microsurgical spinal cord regeneration.
